# Accuracy of Three Commercial Wearable Devices for Sleep Tracking in Healthy Adults

**DOI:** 10.3390/s24206532

**Published:** 2024-10-10

**Authors:** Rebecca Robbins, Matthew D. Weaver, Jason P. Sullivan, Stuart F. Quan, Katherine Gilmore, Samantha Shaw, Abigail Benz, Salim Qadri, Laura K. Barger, Charles A. Czeisler, Jeanne F. Duffy

**Affiliations:** 1 Division of Sleep and Circadian Disorders, Brigham and Women’s Hospital, Boston, MA 02115, USA; mdweaver@mgb.org (M.D.W.); jpsullivan@bwh.harvard.edu (J.P.S.); squan@bwh.harvard.edu (S.F.Q.); kgilmore9@bwh.harvard.edu (K.G.); sshaw0@bwh.harvard.edu (S.S.); abenz@bwh.harvard.edu (A.B.); sqadri@bwh.harvard.edu (S.Q.); lbarger@mgb.org (L.K.B.); cczeisler@bwh.harvard.edu (C.A.C.); jduffy@bwh.harvard.edu (J.F.D.); 2 Division of Sleep Medicine, Harvard Medical School, Boston, MA 02115, USA

**Keywords:** Oura ring, Fitbit, Apple Watch, consumer sleep tracking devices, polysomnography, validation, sleep technology

## Abstract

Sleep tracking by consumers is becoming increasingly prevalent; yet, few studies have evaluated the accuracy of such devices. We sought to evaluate the accuracy of three devices (Oura Ring Gen3, Fitbit Sense 2, and Apple Watch Series 8) compared to the gold standard sleep assessment (polysomnography (PSG)). Thirty-five participants (aged 20–50 years) without a sleep disorder were enrolled in a single-night inpatient study, during which they wore the Oura Ring, Fitbit, and Apple Watch, and were monitored with PSG. For detecting sleep vs. wake, the sensitivity was ≥95% for all devices. For discriminating between sleep stages, the sensitivity ranged from 50 to 86%, as follows: Oura ring sensitivity 76.0–79.5% and precision 77.0–79.5%; Fitbit sensitivity 61.7–78.0% and precision 72.8–73.2%; and Apple sensitivity 50.5–86.1% and precision 72.7–87.8%. The Oura ring was not different from PSG in terms of wake, light sleep, deep sleep, or REM sleep estimation. The Fitbit overestimated light (18 min; *p* < 0.001) sleep and underestimated deep (15 min; *p* < 0.001) sleep. The Apple underestimated the duration of wake (7 min; *p* < 0.01) and deep (43 min; *p* < 0.001) sleep and overestimated light (45 min; *p* < 0.001) sleep. In adults with healthy sleep, all the devices were similar to PSG in the estimation of sleep duration, with the devices also showing moderate to substantial agreement with PSG-derived sleep stages.

## 1. Introduction

Sleep is a complex physiological process, characterized by different stages, each with unique patterns of bodily and brain activity [1]. Healthy sleep, including sufficient duration, quality, and consistently timed sleep, is important for a wide variety of health outcomes, ranging from mental/emotional well-being to cardiovascular, metabolic, and cognitive functions [2,3,4]. Despite the importance of sleep for health and well-being, only one in three US adults report regularly obtaining sufficient sleep duration [5]. Moreover, only 3 in 10 adults in a representative sample in the United States (US) report that their sleep is restorative [6].

There has been an explosion in interest in tracking sleep in the general population. Specifically, nearly one in three Americans reported using their smartphone to track or monitor their sleep in a nationally representative study conducted in 2019, which would equate to approximately 110 million adults [7]. Early consumer-facing sleep monitoring technologies relied on measures such as accelerometry [8], but recent years have seen tremendous advances in the technology. Sensing technologies are increasingly small and increasingly accurate and are capable of sensing movement, temperature, heart rate, and changes in peripheral circulation (i.e., photoplethysmography) [9,10]. Although these advances have the potential to accurately measure sleep, previous research suggests that providing inaccurate or false information about sleep to users (e.g., an inflated number of nighttime awakenings or shorter times than the actual sleep duration) can result in undue stress and worry, and even unnecessary healthcare expenditure [11]. Therefore, ensuring that the data from sleep trackers are accurate and valid is essential to advance the population’s sleep health.

The gold standard measurement of sleep, polysomnography (PSG), includes a range of signals, including brain activity (EEG), eye movements, muscle activity, blood pressure, heart rate, and more [1]. PSG is typically collected in a controlled laboratory or clinic environment, which may have limited generalizability to the home environment. Once collected, the interpretation of PSG includes a skilled technician who scores each 30 s epoch to confirm the changes in physiological signals that characterize different sleep stages [1]. Therefore, while a robust measure of sleep, PSG is time- and resource-intensive and may not reflect sleep in a patient’s home environment. Moreover, inter-rater reliability between technicians scoring PSG data can be variable (research has shown ~80% agreement between two independent PSG interpreters) [12]. Wrist-worn actigraphy is an alternative to PSG commonly used in research that relies on accelerometry to distinguish sleep from wakefulness [13]. Wrist-worn actigraphy has been shown to distinguish well between sleep and wake (between 70 and ~90% agreement with PSG); yet, it has been shown to over-estimate sleep time [14]. The substantial interest among consumers in sleep monitoring (i.e., the fact that more than 100 million adults in the US report using a device to track their sleep) and the promise of increasingly small and accurate devices available to consumers contrast with the limitations of the gold standard measurement. A critical first step in even greater adoption of this technology is ensuring that sleep monitoring devices are providing valid, accurate assessments of sleep and sleep stages to users.

According to a recent report, the Apple Watch is the most commonly used wrist-based device for activity monitoring (58% of individuals who report activity tracking report using an Apple Watch), followed by the Fitbit (25% of those who report activity tracking report using a Fitbit) [15]. Here, we compare these two most commonly used wrist-worn sleep trackers (Apple Watch Series 8 and Fitbit Sense 2) to the most popular ring-based sleep monitoring device (Oura Ring Gen3) [16]. In doing so, we contribute to the growing literature exploring the performance of consumer wearables for sleep tracking [17,18,19].

## 2. Method

### 2.1. Participants

Thirty-five healthy adult participants were recruited through an online study recruitment website at Mass General Brigham to undergo an inpatient PSG. The eligibility criteria included a self-reported sufficient habitual sleep duration between 6 and 9 h, which was confirmed with actigraphy during the week leading into their inpatient study; habitual bedtimes between 9 p.m. and 2 a.m.; body mass index between 18.5 kg/m^2^ and 29.9 kg/m^2^; and agreement to abstain from alcohol, nicotine, and cannabis in the week prior to the inpatient study and to abstain from caffeine in the 2 days prior to the inpatient study. To ensure recruitment of healthy adults, the exclusion criteria included a positive result on validated screening instruments for a sleep disorder, including insomnia, sleep apnea, narcolepsy, sleep apnea, periodic limb movement disorder, nocturnal paroxysmal dystonia, REM sleep behavior disorder, restless legs syndrome, circadian rhythm disorder; current or prior diagnosis of a mental health disorder (e.g., bipolar disorder); pregnancy; presence of caffeine in toxicologic screening; report of an active or uncontrolled medical condition; and having vital signs (heart rate, respiratory rate, blood pressure, temperature) outside normal clinical limits.

### 2.2. Protocol

The participants completed a baseline visit, during which time they agreed to maintain a minimum sleep duration of 8 h nightly. The participants received an activity monitor to be worn continuously for one week prior to their study to confirm their compliance with the sleep duration requirement and bedtime eligibility criteria. In addition, vitals were obtained upon admission for the overnight sleep recording to ensure that blood pressure, heart rate, respiratory rate, and temperature were within healthy ranges. At baseline, the participant’s dorsal skin tone was noted by matching to a visual aid with six skin types (shown in the Supplement), ranging from Type 1 (pale white skin, sensitive to sun) to Type 6 (dark brown to black skin that never burns and is deeply pigmented) [20]. The participants also provided a urine sample for toxicologic and pregnancy testing. One week later, the participants returned their actigraphy device and completed another urine sample. If their actigraphy and urine screen results subsequently indicated eligibility (sleep duration/timing and toxicologic criteria satisfied), they were admitted to the Brigham and Women’s Hospital Center for Clinical Investigation for an overnight inpatient research study. PSG, the Oura Ring (Gen3, worn on the index digit of the non-dominant hand, running the Oura Sleep Staging Algorithm 2.0), the Fitbit Sense 2, worn on one wrist, and the Apple Watch (Series 8), worn on the other wrist, were recorded. All the devices were set to initiate and terminate data collection for an 8 h sleep episode, scheduled according to the average of the participant’s lights off and on times from the prior week.

### 2.3. Polysomnography

Overnight PSG recordings were collected using Vitaport 3 EEG Recorders. The inputs included the electroencephalogram (EEG: F3, F4, C3, C4, O1, O2); electrooculogram (EOG, left outer canthus (LOC) and right outer canthus (ROC)); electrocardiogram (ECG, 2-lead, below the right clavicle at the midclavicular line and on the lower left chest at the anterior axillary line in the 6th or 7th intercostal space); and chin electromyogram (EMG). Sleep stages were scored in 30 s epochs, in accordance with the American Academy of Sleep Medicine scoring guidelines (sampling rate 256 Hz, low-frequency filter 0.159, high-frequency filter 70 Hz) [1].

### 2.4. Data

Data from each participant were aligned by time to facilitate comparison of matched 30 s epochs across devices (epoch-by-epoch analyses). Data from the Oura Ring were obtained from the company in 30 s epochs. Native output from the Apple Watch was obtained in 1 min epochs that we bisected into 30 s epochs to align with the other devices. Output from each device was harmonized into the following categories: sleep, wake, light sleep (PSG stage N1 and N2 sleep), deep sleep (PSG stage N3 sleep), and REM (PSG stage REM sleep). We used the Fitbit sleep stage rather than the Fitbit sleep level after review of the data and supporting Fitbit documentation. The Apple Watch’s “Core” sleep was considered light sleep.

Sleep latency was defined as the elapsed time from lights out to the first epoch of any stage of sleep. Wake after sleep onset (WASO) was defined as all epochs of wake occurring after the first sleep epoch. Sleep efficiency was calculated as the proportion of the recording (from lights out to lights on) spent in any stage of sleep. Total sleep time was calculated by summing all sleep epochs of any sleep stage. Total wake duration was similarly calculated by summing all epochs of wake. Total duration for each sleep stage was also calculated by summing all epochs of each sleep stage. Epoch-by-epoch agreement and duration for each sleep characteristic were estimated for each participant. All durations are reported in minutes.

### 2.5. Wake Interpolation

Across the three devices the duration of the sleep episode from the wearable devices varied somewhat from the actual lights out to the lights on of the PSG-designated sleep episode. The devices were worn and properly turned on; yet, the device-estimated sleep interval could be shorter than the PSG-defined time in bed. Such missing epochs were treated as wake in the present analysis if not detected as part of a sleep episode by a device. Sensitivity analyses comparing findings with and without wake interpolation are included in the Supplementary Materials.

### 2.6. Statistical Analysis

Epoch-by-epoch analyses were conducted in a two-stage classification comparing sleep (yes/no) and wake (yes/no), and also in a four-stage classification comparing wake and light, deep, and REM sleep. Sensitivity, or the proportion within each PSG determination where each device agreed, was calculated as true positive (*tp*)/(*tp* + false negative, *fn*):

$$\frac{tp}{tp+fn}$$

Precision, or positive predictive value, referring to the proportion of device determinations that were confirmed by PSG, was calculated as = *tp*/(*tp* + false positive, *fp*):

$$\frac{tp}{tp+fp}$$

We calculated Kappa to characterize overall agreement between PSG and each device (Oura Ring, Fitbit, and Apple Watch) [21]. Kappa values less than or equal to 0 indicate no agreement; values between 0.01 and 0.20 indicate no to slight agreement; values between 0.21 and 0.40 indicate fair agreement; values between 0.41 and 0.60 indicate moderate agreement; values between 0.61 and 0.80 indicate substantial agreement; and values between 0.80 and 1.00 indicate perfect agreement [21]. Bland–Altman plots were generated to estimate the mean bias and the 95% confidence interval of agreement and to illustrate how agreement varies depending on the mean of the two measurements. These plots present the differences between each device and PSG on the *y*-axis and the mean of each device and PSG on the *x*-axis. Intraclass correlation coefficients (ICCs) were calculated using two-way random effects models; we report the single measure absolute agreement [22]. Concordance is deemed poor if the ICC is below 0.40, fair if between 0.40 and 0.59, good if between 0.60 and 0.74, and excellent if above 0.75 [23]. Paired *t*-tests were used to compare nightly sleep measures for each device to PSG. The assumptions associated with parametric tests were evaluated and confirmed. *p*-values less than 0.05 were considered statistically significant. The analyses were conducted using Stata SE V 15.1 (College Station, TX, USA).

## 3. Results

With respect to age, 40.0% of the participants were 20–29 years, 37.1% were 30–39 years, and 22.9% were 40–50 years of age. Sex distribution was 57% female and 43% male. Racial/ethnic composition was 22.9% Asian, 8.6% Black or African American, 57.1% white, 2.9% more than one race, and 8.6% who preferred not to answer. The participants also represented a variety of skin tones (39% of the sample represented Types 1 through 3, 44% of the sample represented Type 4, and 14% of the sample represented Type 5). See Table 1.

Oura Ring data were obtained for all the participants. The Fitbit failed to record any data for two participants, despite being charged, synced, and initialized. Similarly, there were six participants for whom no Apple data were available, despite the devices being charged, synced, and initialized prior to the participant wearing the device.

### 3.1. Sleep–Wake Agreement

The agreement between PSG and the Oura Ring for sleep–wake was 92.0% of all 30 s epochs in a two-stage (sleep vs. wake) agreement approach (Kappa = 0.60; *p* < 0.001) and 76.3% of all 30 s epochs in a four-stage agreement approach (Kappa = 0.65; *p* < 0.001). PSG and the Fitbit agreed for 91% of all 30 s epochs in a two-stage agreement approach (Kappa = 0.52) and 70.9% of all epochs in a four-stage agreement approach (Kappa = 0.55). PSG-assessed sleep–wake and the Apple Watch agreed for 93% of all 30 s epochs (Kappa = 0.60) and 75.0% of all epochs in a four-stage agreement approach (Kappa = 0.60). The concordance of the sleep–wake agreement, as measured by the Intraclass Correlation Coefficient (ICC), was good for the Oura Ring [0.74 (0.54–0.86)] and Fitbit [0.56 (0.28–0.76)] and excellent for the Apple Watch [0.85 (0.70–0.93)]. 

### 3.2. Sleep Stage Agreement

#### 3.2.1. Sensitivity

In a binary sleep–wake determination, all the devices exhibited ≥95% sensitivity for detection of sleep (Oura: 95% sensitivity (SD 3%); Fitbit: 95% (SD 3%); Apple 97% (SD 2%). In a four-stage classification that compares assignment to wake, light sleep, deep sleep, and REM sleep, the Oura demonstrated sensitivities of 78.2%, 79.5%, and 76.0% for light, deep and REM sleep, respectively, compared to PSG. The Fitbit demonstrated sensitivities of 78.0%, 61.7%, and 67.3% for light, deep, and REM sleep, respectively, compared to PSG. Finally, the Apple Watch demonstrated sensitivities of 86.1%, 50.5%, and 82.6% for light, deep, and REM sleep, respectively, compared to PSG. See Table 2.

#### 3.2.2. Precision

For the Oura, the mean proportion of device-identified epochs that were confirmed by PSG were 79.5%, 77.0%, and 79.1% for light, deep, and REM sleep, respectively. For the Fitbit, the mean proportion of device-identified epochs that were confirmed by PSG were 72.8%, 73.2%, and 73.1% for light, deep, and REM sleep, respectively. For the Apple Watch, the mean proportion of device-identified epochs that were confirmed by PSG were 72.7%, 87.8%, and 77.7% for light, deep, and REM sleep, respectively. See Table 3.

### 3.3. Agreement with Nightly Summary Estimates

**Figure 1:**
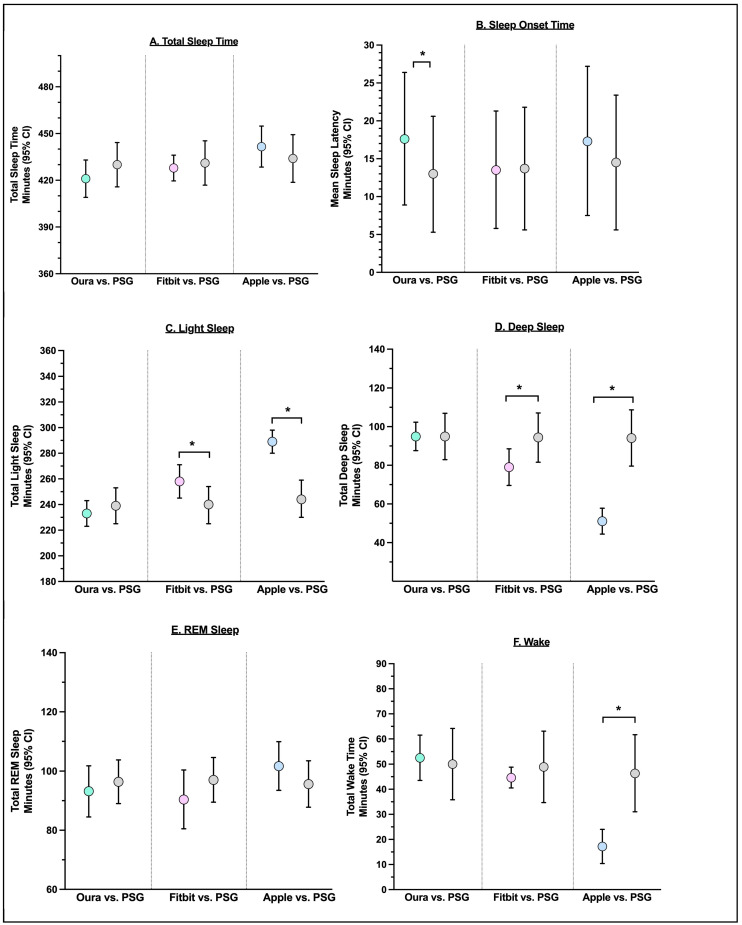
Comparison between each device and PSG ((**A**), sleep efficiency (**B**), wake after sleep onset (WASO, (**C**)), and sleep latency (**D**), REM Sleep (**E**), and Wake (**F**)). Notes. Asterisk (*) indicates *p*-value < 0.05.

The Oura Ring was not significantly different from PSG in the estimation of total sleep time, wake, light sleep, deep sleep, REM, WASO, or sleep efficiency. The Oura Ring significantly overestimated sleep latency compared to PSG by 5 min (*p* < 0.001). The Fitbit significantly overestimated light sleep by 18 min (*p* < 0.001) and underestimated deep sleep by 15 min (*p* < 0.001). The Apple significantly underestimated wake by 7 min (*p* < 0.01), overestimated light sleep by 45 min (*p* < 0.001), underestimated deep sleep by 43 min (*p* < 0.001), and underestimated WASO by 10 min (*p* = 0.02), as compared to PSG. See Table 4 and Figure 1.

**Figure 2:**
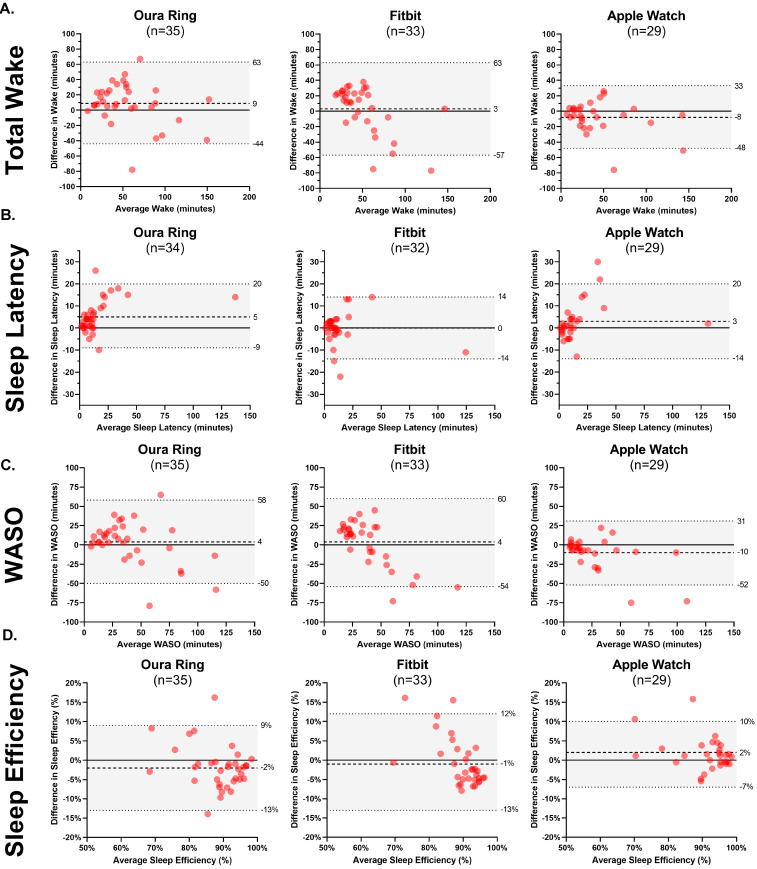
Bland–Altman plots comparing minutes for wake (**A**), sleep latency (**B**), wake after sleep onset (WASO, (**C**)), and sleep efficiency (**D**) between PSG and each device (Oura Ring, Fitbit, Apple Watch).

**Figure 3:**
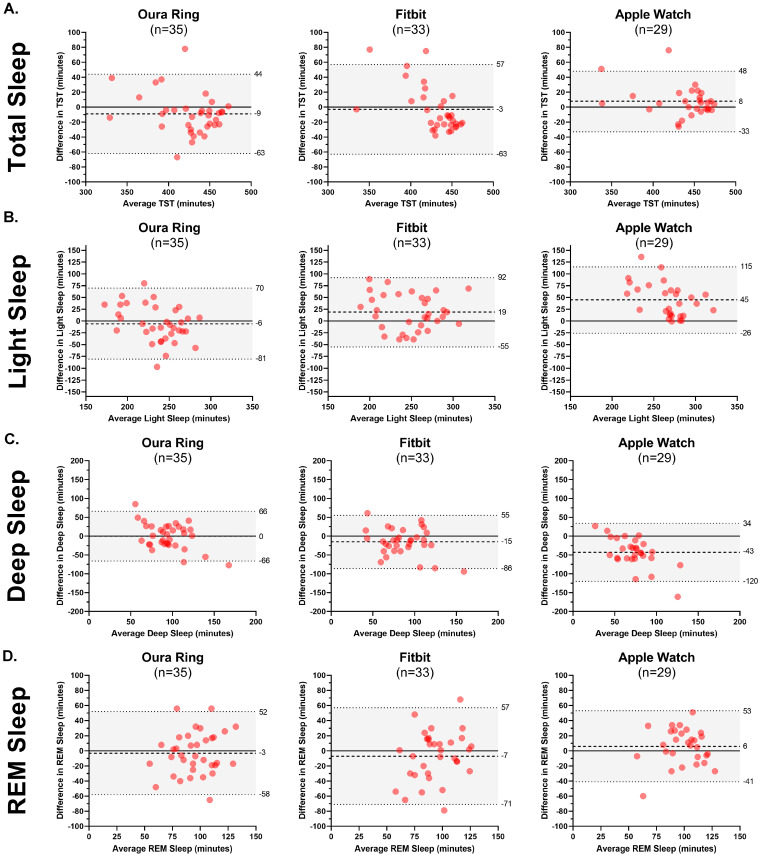
Bland–Altman plots comparing minutes in total sleep (**A**), light sleep (**B**), deep sleep (**C**), and REM sleep (**D**) between PSG and each device (Oura Ring, Fitbit, Apple Watch).

Bland–Altman plots are presented to explore PSG–device ring agreement in greater detail. The mean bias between each device and PSG ranged from 3 min (Fitbit) to 9 min (Oura) for total wake time; between 0 (Fitbit) and 5 min (Oura) for sleep latency; between 4 min (Oura and Fitbit) and 10 min (Apple) for WASO; and between 1% (Fitbit) and 2% (Oura and Apple) for sleep efficiency. See Figure 2. Device–PSG disagreement ranged from 3 min (Fitbit) to 9 min (Oura) for total sleep; between 6 min (Oura) and 45 min (Apple) for light sleep; between 0 min (Oura) and 43 min (Apple) for deep sleep; and between 3 min (Oura) and 7 min (Fitbit) for REM sleep. See Figure 3.

The Oura, Fitbit, and Apple demonstrated between fair and excellent concordance (ICCs ranged from 0.56 to 0.85) with PSG for total sleep time and between poor and fair concordance for light sleep (ICCs ranged from 0.37 to 0.52). The concordance for all the devices was poor for both deep (ICCs ranged from 0.13 to 0.36) and REM sleep (ICCs ranged from 0.13 to 0.37). The devices demonstrated excellent concordance for sleep latency (ICCs ranged from 0.94 to 0.95), from fair to good for WASO (ICCs ranged from 0.41 to 0.72), and from fair to excellent for sleep efficiency (ICCs ranged from 0.56 to 0.85). See Table 5.

**Figure 4:**
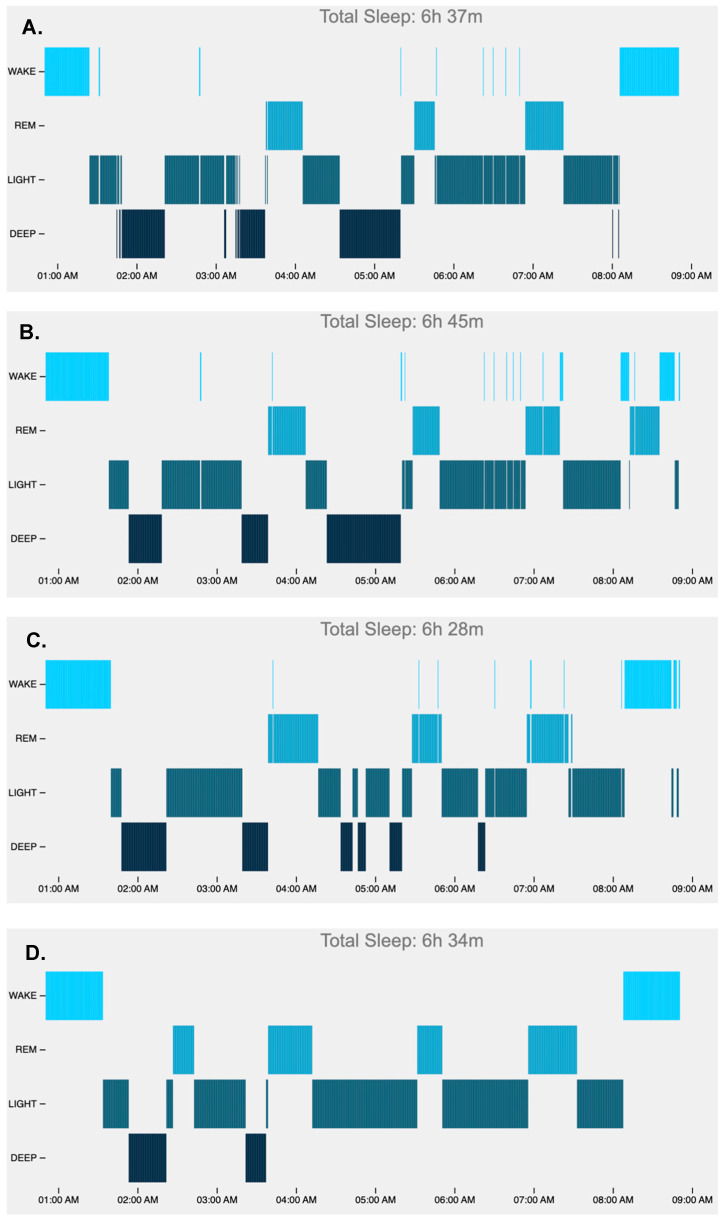
Example hypnograms, including sleep as detected by PSG (**A**), Oura Ring (**B**), Fitbit (**C**), and Apple (**D**).

Example hypnograms from one participant of 30 s epochs comparing PSG with each device evidence a few trends, including the Oura overestimating the initial wake interval, the Fitbit underestimating deep sleep, and the Apple overestimating light and underestimating deep sleep. See Figure 4.

## 4. Discussion

Although the gold standard of sleep assessment (PSG) offers a robust understanding of sleep, the technological advances, such as the increasingly powerful and small nature of sensors, offer promise for the measurement of sleep in naturalistic settings (e.g., a patient’s home), where it can be conducted less intrusively. The results of this validation study of three wearable devices revealed that all the devices exhibited sensitivity of 95% or more for identifying sleep and more than 90% agreement in the determination of sleep vs. wake (i.e., a two-stage classification), surpassing many older research-grade actigraphy devices, which in many cases demonstrated 86–94% agreement with PSG in a two-stage classification [17]. Moreover, our study demonstrated slightly increased rates of agreement compared to the previous research, such as one study that found a sensitivity of 93% for the Oura and 89% for the Fitbit when compared to an ambulatory EEG headband [24]; another study with a similar design (e.g., comparison with ambulatory EEG headband), found a sensitivity of 94% for the Oura and 93% for the Fitbit [25]. Finally, a study comparing the Oura to PSG found 94.5% sensitivity [16].

The Oura Ring exhibited substantial agreement in the determination of specific sleep stages (Kappa > 0.61). The other wearable devices demonstrated moderate agreement (Kappa statistic < 0.61). Epoch-by-epoch analyses demonstrated sensitivity between PSG and the Oura Ring > 75% for all the sleep stages (range: 76.0–79.5%), while the Fitbit varied between 61.7% and 78.0%, and the Apple performed between 50.5% and 86.1% in terms of sensitivity with the PSG sleep stage. The sensitivity between PSG and the Oura is an improvement compared to one of the original validation studies of an earlier model of the Oura Ring, which found between 51% and 65% sensitivity between PSG and the Oura [26].

Of the eight nightly sleep summary characteristics that we examined, the Oura Ring was not significantly different from PSG for seven of the eight measures; the Fitbit provided an overestimate of light sleep and an underestimate of deep sleep. Similarly, the Apple significantly underestimated wake, deep sleep, and WASO and overestimated light sleep. However, with respect to concordance, the ICCs comparing overall nightly estimates for each device-derived sleep parameter to PSG demonstrated poor concordance in the cases of deep sleep and REM sleep. In addition, it is important to note that the Fitbit did not collect data for 2 of the 35 participants enrolled in this study and the Apple did not collect data for 6 of the 35 enrolled participants in this study. In these cases of data loss, the Fitbit and Apple Watch devices had been charged and properly initialized. The Oura recorded data for all the participants on all the study nights.

In several instances, the devices demonstrated satisfactory accuracy, yet comparatively lower concordance. For instance, the Oura Ring exhibited 78.2% and 76.0% sensitivity in detecting deep sleep and REM sleep, respectively; yet, the concordance may be characterized as poor with the PSG-determined nightly totals of deep sleep and REM sleep (ICCs: 0.32 and 0.27, respectively). At the same time, there was no significant difference between these nightly summary estimates when the Oura Ring was compared to PSG using a paired *t*-test. Similarly, the Apple Watch exhibited 50.5% and 82.6% sensitivity in detecting deep sleep and REM sleep, respectively; yet, it showed poor concordance with the PSG-determined deep sleep and REM sleep (ICCs: 0.13 and 0.37, respectively). There are several explanations for these apparent contradictions. First, the accuracy, sensitivity, and precision were from the epoch-by-epoch analyses, while concordance and paired *t*-tests compared the overall nightly estimates of each sleep parameter. Second, although the PSG-determined stage matched the device-determined stage between 51% and 83% of the time, high variability in device misclassification could have contributed to low concordance. ICCs are sensitive to differences in the variability of the raters [22], whereas the paired *t*-tests are more sensitive to differences in the mean estimates. Collectively, those utilizing these wearable devices received estimates of nightly summary measures that were comparable to PSG (e.g., the nightly estimate for total sleep time was within 10 min of PSG for all the devices); however, the nightly estimates for each individual may have diverged more widely (Figure 2). Finally, research has shown that even trained human coders achieve only approximately 80% correlation with one another, and the majority of errors made by these human coders are typically in the transitions between REM and other stages [13]. Therefore, the reliability of even trained and experienced human coders may only be moderate for scoring stages such as REM.

Whereas previous studies relied on young populations (e.g., average age 20–30 years [27]), a strength of our study is the diversity of age (20 to 50 years old). Our sample was also diverse in terms of race/ethnicity, as more than 30% of the sample comprised Asian, Black, or African American participants or individuals who identified as more than one race. Diversity in skin tone is particularly important for consumer sleep tracking devices that utilize photoplethysmography, given that research has demonstrated varied signal densities among individuals with darker skin tones [28].

## 5. Conclusions

Consumer sleep technologies hold tremendous potential as robust, low-cost approaches to sleep measurement. Moreover, through the lens of behavioral theory, which emphasizes self-monitoring as a key component of adherence to any behavioral regimen [29], accurate sleep measurement could provide ongoing motivation to improve sleep routines and habits. Our analyses revealed that all three devices perform extremely well in distinguishing between sleep and wake. Future research should explore the performance of the devices (as well as other devices not tested in the current study) in other populations, such as those with poor sleep, nightshift workers who have to sleep during the day, and/or those with sleep disorders. It is likely that such individuals may be seeking solutions to measure and/or improve their sleep, such as sleep monitoring devices. Ensuring that consumer devices perform well in populations with disrupted sleep, not just in individuals with healthy sleep, is imperative, to attenuate the risk of undue stress, worry, and unnecessary healthcare expenditure, when the data available to users are inaccurate [11]. Future research should also explore the performance of the heart rate variability indices available on wearable devices, such as the Oura, in comparison to the gold standard measures. It is important to note that the cost of these devices remains high (range: USD 249.99 to USD 799.00) and out of reach for many individuals.

A limitation is that only a single night of data was collected, and the devices were only compared to PSG during scheduled sleep episodes in healthy participants rather than across a 24 h interval, which is the way most wearables are used. This overestimates concordance, since wearables erroneously score quiet wakefulness (e.g., while watching a movie or reading a book during the daytime) and the analysis was restricted to a limited time within the 24-h day (the scheduled sleep episode) when sleep was highly likely to occur. Future research should evaluate device performance across multiple nights. Moreover, studying healthy adults without insomnia further limited generalizability, since sleep was very likely to occur during the analysis window in healthy adults. In addition, we were unable to collect Fitbit and Apple Watch data from several participants because of device failure. There are a large and growing number of wearables on the market, but we used only three devices; therefore, our findings are not generalizable to other devices or subsequent models of the tested devices. Future research should consider a comparison of all devices with PSG in order to allow the consumer to select the best device for their needs.

It is a major limitation that the vast majority of wearable companies neither provide access to raw data collected from the sensors in their devices nor provide insight into the algorithms used to score signal data [27]. As argued by de Zambotti and colleagues in their paper “State of the science and recommendations for using wearable technology in sleep and circadian research”, the failure of wearable device manufacturers to provide access to raw data derived from their sensors or guidance on their algorithms precludes analyses of sensor performance from the standpoint of sensor technology and scoring algorithms. Furthermore, the ‘black-box’ nature of sensor data and algorithms hinders the reproducibility of research with wearables [27]. In addition, our participants were kept in bed for 8 h at night; so, it is unclear how the Oura, Fitbit and Apple Watch perform during long or short sleep episodes or during daytime sleep (e.g., naps) and quiet wakefulness. Moreover, the controlled laboratory setting is probably not generalizable to sleep in the home environment. Our primary analysis assigned intervals of wake for each device when the device did not detect a sleep episode. Sensitivity analyses (Supplement) confirm similar results without wake interpolation overall and when participants with ≥5 min of missing data are excluded.

## Figures and Tables

**Table 1:** Demographic characteristics of the study sample (n = 35).

Characteristic		n	%
Age	20 to 29 years of age	14	40.0%
	30 to 39 years of age	13	37.1%
	40 to 50 years of age	8	22.9%
Sex	Female	20	57.1%
	Male	15	42.9%
Race/Ethnicity	White, non-Hispanic	20	57.1%
	Asian	8	22.9%
	Black or African American	3	8.6%
	More than one race	1	2.9%
	Preferred not to answer	3	8.6%
Skin Type	Type 1	1	2.9%
	Type 2	1	2.9%
	Type 3	12	34.3%
	Type 4	16	45.7%
	Type 5	5	14.2%

**Table 2:** Sensitivity: mean and standard deviation of epochs assigned to each sleep stage by each wearable and PSG. Sensitivity was calculated as true positive/(true positive + false negative).

Oura Assignment	PSG Assignment			
	Wake	Light	Deep	REM
Wake	68.6% (18.4%)	7.4% (4.6%)	0.1% (0.2%)	5.8% (7.3%)
Light	19.0% (11.6%)	78.2% (7.6%)	19.9% (14.1%)	17.8% (13.1%)
Deep	2.6% (3.6%)	8.5% (6.5%)	79.5% (14.5%)	0.4% (0.9%)
REM	9.7% (10.9%)	6.0% (5.0%)	0.5% (1.6%)	76.0% (15.2%)
**Fitbit Assignment**	Wake	Light	Deep	REM
Wake	67.7% (23.1%)	6.8% (4.2%)	1.5% (1.6%)	6.1% (4.5%)
Light	23.3% (18.8%)	78.0% (9.0%)	34.8% (20.4%)	24.5% (20.9%)
Deep	0.8% (2.9%)	7.5% (5.8%)	61.7% (21.1%)	1.8% (5.3%)
REM	8.2% (15.3%)	7.7% (6.6%)	2.1% (6.9%)	67.6% (23.3%)
**Apple Assignment**	Wake	Light	Deep	REM
Wake	52.4% (21.5%)	4.2% (3.5%)	0.3% (0.7%)	1.4% (2.0%)
Light	37.2% (18.2%)	86.1% (6.2%)	48.3% (20.0%)	15.8% (14.4%)
Deep	1.0% (1.9%)	2.1% (3.2%)	50.5% (20.6%)	0.2% (1.1%)
REM	9.4% (12.8%)	7.6% (4.7%)	0.9% (2.0%)	82.6% (14.8%)

Note: Sensitivity for each cell was calculated at the participant level. Mean and standard deviation of participant-level estimates are presented. Estimates represent 35 participants for Oura, 33 participants for Fitbit, and 29 participants for Apple.

**Table 3:** Precision: mean and standard deviation of epochs assigned to each sleep stage by each wearable and PSG. Precision or positive predictive value was calculated as = true positive/(true positive + false positive).

Oura Assignment	PSG Assignment			
	Wake	Light	Deep	REM
Wake	53.5% (24.4%)	35.4% (21.1%)	0.4% (1.0%)	10.7% (11.8%)
Light	3.7% (3.9%)	79.5% (8.6%)	9.1% (8.6%)	7.6% (5.7%)
Deep	0.8% (0.7%)	21.5% (17.0%)	77.0% (17.4%)	0.5% (0.9%)
REM	5.9% (8.6%)	14.5% (11.2%)	0.5% (1.4%)	79.1% (13.1%)
**Fitbit Assignment**	Wake	Light	Deep	REM
Wake	51.0% (21.7%)	33.9% (18.0%)	3.1% (3.3%)	12.0% (10.1%)
Light	5.6% (7.9%)	72.8% (13.4%)	12.8% (9.8%)	8.8% (6.5%)
Deep	1.3% (5.9%)	23.3% (18.6%)	73.2% (21.5%)	1.8% (5.1%)
REM	4.5% (9.5%)	20.3% (18.3%)	2.1% (6.5%)	73.1% (21.9%)
**Apple Assignment**	Wake	Light	Deep	REM
Wake	61.9% (23.0%)	33.4% (22.8%)	0.8% (2.4%)	3.9% (4.8%)
Light	5.4% (5.5%)	72.7% (10.3%)	16.5% (11.1%)	5.5% (5.2%)
Deep	0.6% (0.9%)	10.4% (17.3%)	87.8% (19.7%)	0.6% (2.8%)
REM	3.6% (5.3%)	17.8% (10.5%)	0.9% (2.2%)	77.7% (12.9%)

Note. Precision for each cell was calculated at the participant level. Mean and standard deviation of participant-level estimates are presented here. Estimates represent 35 participants for Oura, 33 participants for Fitbit, and 29 participants for Apple.

**Table 4:** Comparison of Oura, Fitbit, and Apple Watch to PSG with respect to sleep/wake and sleep stages.

	Oura n = 35 *Mean* (*SD*)	PSG n = 35 *Mean* (*SD*)	Fitbit n = 33 *Mean* (*SD*)	PSG n = 33 *Mean* (*SD*)	Apple Watch n = 29 *Mean* (*SD*)	PSG n = 29 *Mean* (*SD*)
Total Sleep Time (min)	421 (34)	430 (41)	428 (23)	431 (40)	442 (35)	434 (40)
Wake (min)	59 (34)	50 (41)	49 (26)	49 (40)	39 (35) *	46 (40)
Light Sleep (min)	233 (28)	239 (41)	258 (37) *	240 (41)	289 (24) *	244 (38)
Deep Sleep (min)	95 (21)	95 (35)	79 (27) *	94 (36)	51 (18) *	94 (38)
REM (min)	93 (25)	96 (21)	90 (28)	97 (21)	102 (22)	96 (21)
Sleep Latency (min)	18 (25) *	13 (22)	13 (21)	13 (22)	17 (26)	15 (23)
WASO (min)	42 (26)	38 (37)	40 (15)	36 (35)	22 (23) *	32 (33)
Sleep Efficiency (%)	88% (7%)	90% (9%)	89% (5%)	90% (8%)	92% (7%)	90% (8%)

Notes. Missing data were reassigned as wake (see section on wake interpolation). Sample size for sleep latency n = 34 for PSG vs. Oura, n = 32 for PSG vs. Fitbit, and n = 29 for PSG vs. Apple Watch. * Indicates significant difference between the device and PSG using paired *t*-tests.

**Table 5:** Intraclass correlation coefficients between each device and PSG across characteristic of sleep.

	Oura n = 35	Fitbit n = 33	Apple Watch n = 29
	*ICC* (*95% CI*)	*ICC* (*95% CI*)	*ICC* (*95% CI*)
Total Sleep Time (min)	0.74 (0.54–0.86)	0.56 (0.28–0.76)	0.85 (0.70–0.93)
Light Sleep (min)	0.40 (0.08–0.64)	0.52 (0.22–0.73)	0.37 (0.00–0.64)
Deep Sleep (min)	0.32 (−0.01–0.59)	0.36 (0.02–0.62)	0.13 (−0.24–0.47)
REM (min)	0.27 (−0.06–0.55)	0.13 (−0.22–0.45)	0.37 (0.01–0.64)
Sleep Latency (min)	0.95 (0.90–0.97)	0.95 (0.89–0.97)	0.94 (0.87–0.97)
WASO (min)	0.63 (0.38–0.80)	0.41 (0.08–0.66)	0.72 (0.48–0.86)
Sleep Efficiency (%)	0.74 (0.55–0.86)	0.56 (0.28–0.76)	0.85 (0.71–0.93)

Notes. ICCs represent comparisons between PSG and each device (Oura, Fitbit, and Apple Watch). Sample size for sleep latency is n = 34 for PSG vs. Oura, n = 32 for PSG vs. Fitbit, and n = 29 for PSG vs. Apple Watch. Reliability is deemed ‘poor’ if the ICC is below 0.4, ‘fair’ if between 0.4 and 0.59, ‘good’ if between 0.60 and 0.74, and ‘excellent’ if above 0.75.

## Data Availability

The authors will make de-identified data from the current study available upon written request. Execution of a Materials Transfer Agreement is required if the data are to be used in research supported by a for-profit company, as per Mass General Brigham Institutional Review Board policy.

## Supplementary Materials

The following supporting information can be downloaded at: https://www.mdpi.com/article/10.3390/s24206532/s1, Figure S1: Participants with missing data by time during the overnight recording. Table S1: Secondary Analysis 1. Comparison of Oura, FitBit, and Apple Watch to PSG with respect to sleep/wake and sleep stages (including all participants). Epochs of missing data were excluded from the analysis. Table S2: Secondary Analysis 2. Comparison of Oura, FitBit, and Apple Watch to PSG with respect to sleep/wake and sleep stages after excluding those with >5 min of missing data. Table S3: Epochs assigned to each sleep stage by each wearable and PSG. The total number of epochs is presented, followed by the row percentage, then the column percentage. Total F1 score is inclusive of the 4 categories in this table. F1 score was calculated as: (true positive/(true positive + (0.5*(false positive + false negative)))). Table S4: Epochs assigned to sleep and wake by each device. The total number of epochs is presented, followed by the row percentage, then the column percentage. Total F1 score is inclusive of the 2 categories in this table. References [30,31,32] are cited in the supplementary materials.
