# Supplementary material for: Accuracy of Three Commercial Wearable Devices for Sleep Tracking in Healthy Adults
Source: Sensors (Basel). 2024 Oct 10;24(20):6532. doi: 10.3390/s24206532 (PMC11511193; doi:10.3390/s24206532)
Supplement: Supplementary file 1 [file sensors-24-06532-s001.zip › sensors-3172750-supplementary.pdf]

Supplement Table of Contents

Section	Description	Pages
1.	Fitzpatrick Skin Tone assessment	2
2.	Methodological Approach	3
3.	Sensitivity Analyses	4
4.	Epoch-by-Epoch Assignments	5-6
5.	Additional References	7

Section 1: Details on the visual aid used to ascertain skin tone

Participant skin tone was noted by comparison of the dorsal hand to the below descriptions and visual aids for the 6 skin tones outlined by Fitzpatrick.

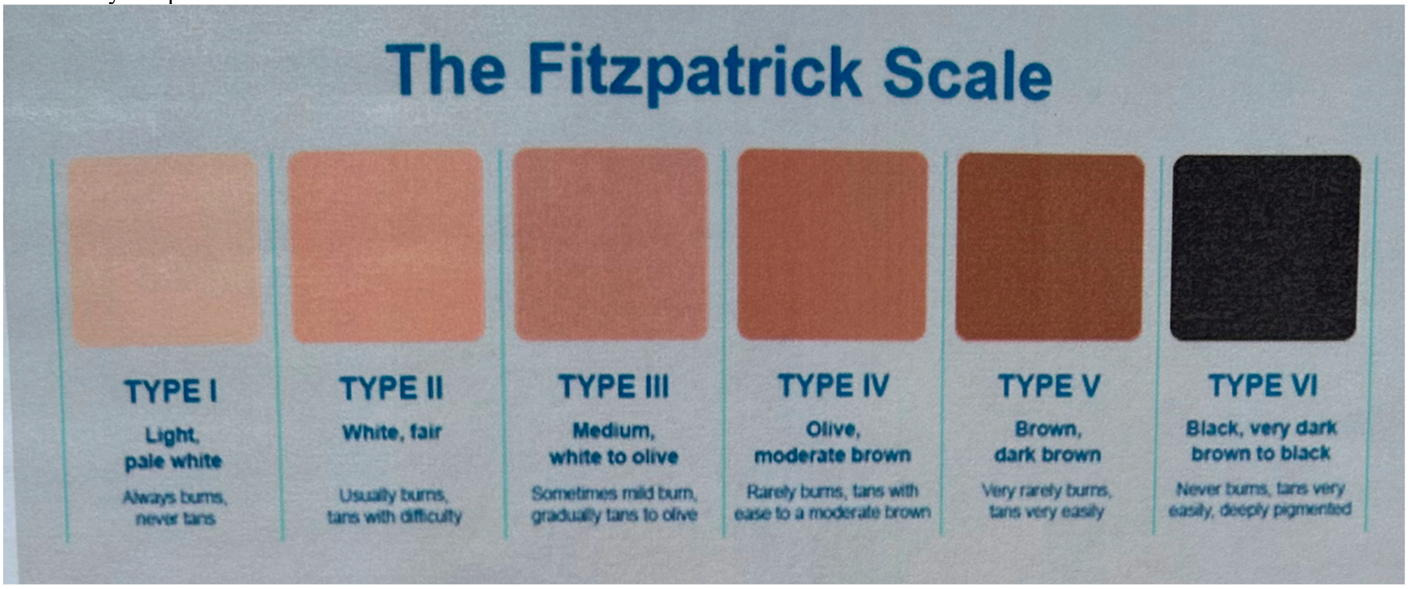

Section 2: Methodological Approach

The Oura ring on each participant's non-dominant hand was used as the primary Oura recording. Twenty-two of the 35 participants had intervals of incomplete data on the Oura recording. These were nearly always concentrated at the beginning of the recording and most often lasted less than 5 minutes. Of the 33 participants with FitBit data, 14 had at least one epoch with missing/undefined data. The median duration of undefined data for FitBit was 0 minutes (IQR 0-3.5). Of the 29 participants with Apple Watch data, 26 had intervals of missing/undefined data throughout the night. The median duration of undefined data for Apple Watch was 6.5 minutes (IQR 10.5-20.5). Across devices, these intervals of missing data tended to occur at the beginning or end of the recording (illustrated in Exhibit 1 below).

Figure S1. Participants with missing data by time during the overnight recording.

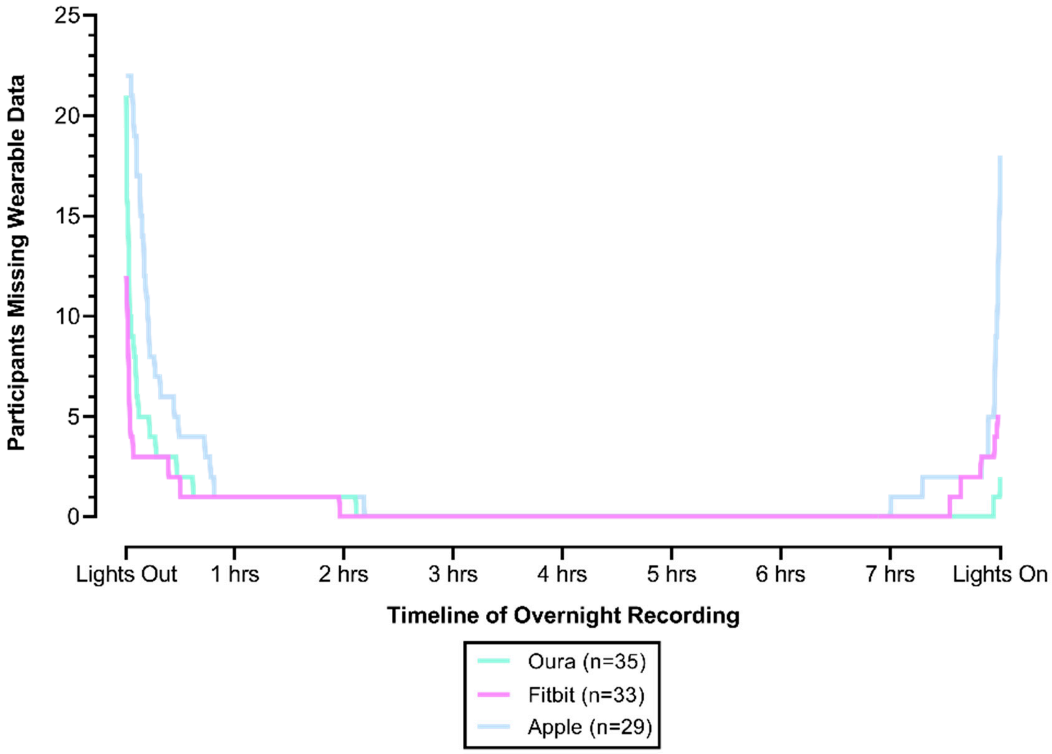

After consideration of the device methodology and a review of the literature, we reassigned these intervals of missing data as Wake for the primary analysis. The same procedure was followed across all devices for consistency. These intervals of missingness were not due to connectivity or other issues. Thus, we concluded that the devices did not assign wake/sleep because the device did not identify the interval as a potential sleep interval. The primary analysis includes complete data for all devices assuming these intervals are wake. We also carried out two secondary analytic approaches: 1) we present the findings without reassignment of missing data; and 2) we exclude participants with more than 5 minutes of missing data. All of these approaches have been implemented by other investigators in this space,[1-6] with the threshold for exclusion of missing data varying (e.g. 10 minutes).[2]

Section 3: Sensitivity Analyses

The following tables present point estimates for sleep characteristics as estimated by each device and their matched PSG comparators. Significant differences between each device and PSG for that characteristic of sleep is indicated with an *. Table 1 presents secondary analyses of these data without wake-padding (missing data are considered missing). Table 2 presents these data among the subset of participants with <5 minutes of missing data.

Table S1. Secondary Analysis 1. Comparison of Oura, FitBit, and Apple Watch to PSG with respect to sleep/wake and sleep stages (including all participants). Epochs of missing data were excluded from the analysis.

	Oura n=35 Mean (SD)	PSG n=35 Mean (SD)	FitBit n=33 Mean (SD)	PSG n=33 Mean (SD)	Apple Watch n=29 Mean (SD)	PSG n=29 Mean (SD)
Total Sleep Time (min)	421 (34)	430 (41)	428 (23)	431 (40)	442 (35)	434 (40)
Wake (min)	53 (26)	50 (41)	45 (12)	49 (40)	17 (18)*	46 (40)
Light Sleep (min)	233 (28)	239 (41)	258 (37)*	240 (41)	289 (24)*	244 (38)
Deep Sleep (min)	95 (22)	95 (35)	79 (27)*	94 (36)	51 (18)*	94 (38)
REM (min)	93 (25)	96 (21)	90 (28)	97 (21)	102 (22)	96 (21)
Sleep Latency (min)**	10 (7)	13 (22)	7 (6)	14 (22)	0 (1)	15 (23)
WASO (min)	42 (26)	38 (37)	38 (13)	36 (35)	17 (18)*	32 (33)
Sleep Efficiency (%)	89% (6%)	90% (9%)	91% (2%)	90% (8%)	96% (4%)*	90% (8%)

**Sample size is n=34 for PSG vs. Oura, n=32 for PSG vs. FitBit, and n=29 for PSG vs. Apple Watch.
*Indicates significant difference between the device and PSG using paired t-tests.

Table S2. Secondary Analysis 2. Comparison of Oura, FitBit, and Apple Watch to PSG with respect to sleep/wake and sleep stages after excluding those with >5 minutes of missing data.

	Oura n=27 Mean (SD)	PSG n=27 Mean (SD)	FitBit n=27 Mean (SD)	PSG n=27 Mean (SD)	Apple Watch n=4 Mean (SD)	PSG n=4 Mean (SD)
Total Sleep Time (min)	430 (27)	439 (34)	435 (13)	442 (27)	458 (22)	455 (18)
Wake (min)	49 (27)	41 (34)	44 (12)	38 (27)	21 (23)	26 (18)
Light Sleep (min)	237 (28)	241 (40)	263 (36)*	248 (38)	298 (21)	259 (14)
Deep Sleep (min)	97 (22)	97 (26)	81 (28)	95 (35)	56 (15)*	96 (4)
REM (min)	96 (26)	101 (22)	91 (29)	99 (21)	105 (15)	100 (13)
Sleep Latency (min)**	9 (7)	7 (5)	7 (6)	8 (4)	2 (2)	4 (4)
WASO (min)	40 (26)	34 (35)	37 (26)	31 (28)	19 (20)	22 (21)
Sleep Efficiency (%)	90% (6%)	91% (7%)	91% (2%)	92% (6%)	96% (4%)	95% (4%)

**Sample size is n=27 for PSG vs. Oura, n=26 for PSG vs. FitBit, and n=4 for PSG vs. Apple Watch.
*Indicates significant difference between the device and PSG using paired t-tests.

Section 4: Assignment of all epochs across participants by each device with additional measures of agreement.

The primary analyses presented in the main document calculate precision and sensitivity for each participant and then average these measures of agreement across the 35 nights of recording for each sleep characteristic. This section displays the assignment of all epochs across participants in a 4-stage and 2-stage agreement format.

Table S4. Epochs assigned to each sleep stage by each wearable and PSG. The total number of epochs is presented, followed by the row percentage, then the column percentage. Total F1 score is inclusive of the 4 categories in this table. F1 score was calculated as: (true positive / (true positive + (0.5*(false positive + false negative)))).

	PSG Assignment					F1 Score
**Oura Assignment**	Wake	Light	Deep	REM	Total	
Wake<br>Row % (Precision)<br>Col % (Sensitivity)	2,478<br>59.8%<br>70.8%	1,271<br>30.7%<br>7.6%	11<br>0.3%<br>0.2%	384<br>9.3%<br>5.7%	4,144<br>100%<br>12.3%	**0.65**
Light<br>Row % (Precision)<br>Col % (Sensitivity)	596<br>3.7%<br>17.0%	12,976<br>79.6%<br>77.7%	1,495<br>9.2%<br>22.5%	1238<br>7.6%<br>18.4%	16,305<br>100%<br>48.5%	**0.79**
Deep<br>Row % (Precision)<br>Col % (Sensitivity)	56<br>0.84%<br>1.60%	1,445<br>21.8%<br>8.7%	5,103<br>76.9%<br>76.8%	29<br>0.4%<br>0.4%	6,633<br>100%<br>19.7%	**0.77**
REM<br>Row % (Precision)<br>Col % (Sensitivity)	372<br>5.7%<br>10.6%	1,018<br>15.6%<br>6.1%	34<br>0.5%<br>0.5%	5,097<br>78.2%<br>75.5%	6,521<br>100%<br>19.4%	**0.77**
Total<br>Row %<br>Col %	3,502<br>10.4%<br>100%	16,710<br>49.7%<br>100%	6,643<br>19.8%<br>100%	6,748<br>20.1%<br>100%	33,603<br>100%<br>100%	**0.76**
**Fitbit Assignment**	Wake	Light	Deep	REM	Total	
Wake<br>Row % (Precision)<br>Col % (Sensitivity)	1,908<br>55.3%<br>59.1%	1,069<br>31.0%<br>6.8%	93<br>2.7%<br>1.5%	1,908<br>55.3%<br>59.1%	3,453<br>100%<br>10.9%	**0.57**
Light<br>Row % (Precision)<br>Col % (Sensitivity)	947<br>5.6%<br>29.3%	12,379<br>72.6%<br>78.3%	2,159<br>12.7%<br>34.7%	1,570<br>9.2%<br>24.5%	17,055<br>100%<br>53.8%	**0.75**
Deep<br>Row % (Precision)<br>Col % (Sensitivity)	69<br>1.3%<br>2.1%	1,172<br>22.5%<br>7.4%	3,846<br>73.9%<br>61.8%	118<br>2.3%<br>1.8%	5,205<br>100%<br>16.4	**0.67**
REM<br>Row % (Precision)<br>Col % (Sensitivity)	306<br>5.1%<br>9.5%	1,199<br>20.1%<br>7.6%	130<br>2.2%<br>2.1%	4,333<br>72.6%<br>67.7%	5,968<br>100%<br>18.8%	**0.70**
Total<br>Row %<br>Col %	3,230<br>10.2%<br>100%	15,819<br>49.9%<br>100%	6,228<br>19.7%<br>100%	6,404<br>20.2%<br>100%	31,681<br>100%<br>100%	**0.71**
**Apple Assignment**	Wake	Light	Deep	REM	Total	
Wake<br>Row % (Precision)<br>Col % (Sensitivity)	1,563<br>69.3%<br>58.2%	601<br>26.7%<br>4.2%	17<br>0.8%<br>0.3%	74<br>3.3%<br>1.3%	2,255<br>100%<br>8.1%	**0.63**
Light<br>Row % (Precision)<br>Col % (Sensitivity)	879<br>5.3%<br>32.7%	12,183<br>72.7%<br>86.0%	2,787<br>16.6%<br>51.0%	906<br>5.4%<br>16.3%	16,755<br>100%<br>60.2%	**0.79**
Deep<br>Row % (Precision)<br>Col % (Sensitivity)	18<br>0.6%<br>0.7%	311<br>10.5%<br>2.2%	2,606<br>88.3%<br>47.7%	15<br>0.5%<br>0.3%	2,950<br>100%<br>10.6%	**0.62**
REM<br>Row % (Precision)<br>Col % (Sensitivity)	227<br>3.9%<br>8.5%	1,069<br>18.1%<br>7.6%	50<br>0.9%<br>0.9%	4,551<br>77.2%<br>82.1%	5,897<br>100%<br>21.2%	**0.80**
Total<br>Row %<br>Col %	2,687<br>9.7%<br>100%	14,164<br>50.9%<br>100%	5,460<br>19.6%<br>100%	5,546<br>19.9%<br>100%	27,857<br>100%<br>100%	**0.75**

Table S5. Epochs assigned to sleep and wake by each device. The total number of epochs is presented, followed by the row percentage, then the column percentage. Total F1 score is inclusive of the 2 categories in this table.

	PSG Assignment			F1 Score*
**Oura Assignment**	Wake	Sleep	Total	
Wake<br>Row % (Precision)<br>Col % (Sensitivity)	2,478<br>59.8%<br>70.8%	1,666<br>40.2%<br>5.5%	4,144<br>100%<br>12.3%	**0.65**
Sleep<br>Row % (Precision)<br>Col % (Sensitivity)	1,024<br>3.5%<br>29.2%	28,435<br>96.5%<br>94.5%	29,459<br>100%<br>87.7%	**0.95**
Total<br>Row %<br>Col %	3,502<br>10.4%<br>100%	30,101<br>89.6%<br>100%	33,603<br>100%<br>100%	**0.92**
**Fitbit Assignment**	Wake	Sleep	Total	
Wake<br>Row % (Precision)<br>Col % (Sensitivity)	1,908<br>55.3%<br>59.1%	1,545<br>44.7%<br>5.4%	3,453<br>100%<br>10.9%	**0.57**
Sleep<br>Row % (Precision)<br>Col % (Sensitivity)	1,322<br>4.7%<br>40.9%	26,906<br>95.3%<br>94.6%	28,228<br>100%<br>89.1%	**0.95**
Total<br>Row %<br>Col %	3,230<br>10.2%<br>100%	28,451<br>89.8%<br>100%	31,681<br>100%<br>100%	**0.91**
**Apple Assignment**				
Wake<br>Row % (Precision)<br>Col % (Sensitivity)	1,563<br>69.3%<br>58.2%	692<br>30.7%<br>2.8%	2,255<br>100%<br>8.1%	**0.63**
Sleep<br>Row % (Precision)<br>Col % (Sensitivity)	1,124<br>4.4%<br>41.8%	24,478<br>95.6%<br>97.3%	25,602<br>100%<br>91.9%	**0.96**
Total<br>Row %<br>Col %	2,687<br>9.7%<br>100%	25,170<br>90.4%<br>100%	27,857<br>100%<br>100%	**0.93**

References

1. Altini M, Kinnunen H. The Promise of Sleep: A Multi-Sensor Approach for Accurate Sleep Stage Detection Using the Oura Ring. *Sensors (Basel).* 2021;21(13).
2. Chinoy ED, Cuellar JA, Huwa KE, et al. Performance of seven consumer sleep-tracking devices compared with polysomnography. *Sleep.* 2021;44(5).
3. de Zambotti M, Rosas L, Colrain IM, Baker FC. The Sleep of the Ring: Comparison of the OURA Sleep Tracker Against Polysomnography. *Behav Sleep Med.* 2019;17(2):124-136.
4. Nguyen QNT, Le T, Huynh QBT, Setty A, Vo TV, Le TQ. Validation Framework for Sleep Stage Scoring in Wearable Sleep Trackers and Monitors with Polysomnography Ground Truth. *Clocks Sleep.* 2021;3(2):274-288.
5. Chee NI, Ghorbani S, Golkashani HA, Leong RL, Ong JL, Chee MW. Multi-night validation of a sleep tracking ring in adolescents compared with a research actigraph and polysomnography. *Nature and science of sleep.* 2021:177-190.
6. Svensson T, Madhawa K, Nt H, Chung U-i, Svensson AK. Validity and reliability of the Oura Ring Generation 3 (Gen3) with Oura sleep staging algorithm 2.0 (OSSA 2.0) when compared to multi-night ambulatory polysomnography: A validation study of 96 participants and 421,045 epochs. *Sleep Medicine.* 2024;115:251-263.
7. Fitzpatrick TB. The Validity and Practicality of Sun-Reactive Skin Types I Through VI. *Archives of Dermatology*. 1988;124(6):869-871.
